# Chimera Patterns of Synchrony in a Frustrated Array of Hebb Synapses

**DOI:** 10.3389/fncom.2022.888019

**Published:** 2022-06-23

**Authors:** A. E. Botha, M. Ansariara, S. Emadi, M. R. Kolahchi

**Affiliations:** ^1^Department of Physics, Science Campus, University of South Africa, Private Bag X6, Johannesburg, South Africa; ^2^Department of Physics, Institute for Advanced Studies in Basic Sciences, Zanjan, Iran; ^3^Department of Biological Sciences, Institute for Advanced Studies in Basic Sciences, Zanjan, Iran

**Keywords:** chimera states, Hebb rule, synchronization, spike timing-dependent plasticity, Kuramoto-Sakaguchi model, frustrated coupling

## Abstract

The union of the Kuramoto–Sakaguchi model and the Hebb dynamics reproduces the Lisman switch through a bistability in synchronized states. Here, we show that, within certain ranges of the frustration parameter, the chimera pattern can emerge, causing a different, time-evolving, distribution in the Hebbian synaptic strengths. We study the stability range of the chimera as a function of the frustration (phase-lag) parameter. Depending on the range of the frustration, two different types of chimeras can appear spontaneously, i.e., from randomized initial conditions. In the first type, the oscillators in the coherent region rotate, on average, slower than those in the incoherent region; while in the second type, the average rotational frequencies of the two regions are reversed, i.e., the coherent region runs, on average, faster than the incoherent region. We also show that non-stationary behavior at finite *N* can be controlled by adjusting the natural frequency of a single pacemaker oscillator. By slowly cycling the frequency of the pacemaker, we observe hysteresis in the system. Finally, we discuss how we can have a model for learning and memory.

## 1. Introduction

Brain transplantation of cell-derived cortical pyramidal neurons into the mouse cortex makes for a mouse/human chimera model that helps in the study of human brain development (Linaro et al., 2019; Le Bras, 2020). The chimera model takes its name in a similar manner from Greek mythology (Kuramoto and Battogtokh, 2002; Abrams and Strogatz, 2004).

Even from the mythological standpoint, it is inspiring to think about how this monster can come about. This monster takes its character from collecting facets that come together only in exceptional circumstances.

In physics, when we talk about emergent properties, we know that exceptional circumstances should come together to give birth to such novel traits of beauty. But, beauty or beast, the idea is of the same basic nature (Ikegami et al., 2017). The collective nature of such particular phenomena serves some function and, as the following example indicates, the reductionist approach is insufficient to elucidate its formation.

There is a form of fear, named *sensitization*, that is learned. The learning mechanism leading to it was studied in *Aplysia*, a sea snail, and compared to that of a vertebrate. It was discovered that for the case of learning in snail as well as the complex learning in mammals, the same biochemical mechanism holds—for both, the long–term memory differs from the short–term memory in that, the long–term memory requires the synthesis of new protein (Kandel, 2001).

Chimeras, as a particular coexistence of synchronized and desynchronized states, can help in modeling phenomena ranging from epilepsy (Wang and Liu, 2020; Frolov and Hramov, 2021) to power grid outages (Haugland, 2021; Parastesh et al., 2021). The collective behavior in a multilayer network of coupled neurons is compared to mechanisms in the brain (Majhi et al., 2017). From a neurophysiological standpoint, the chimera state can model the unihemispheric sleep in dolphins, and some other marine mammals (Mukhametov et al., 1977; Rattenborg et al., 2000; Wang and Liu, 2020).

The network we consider in our model, benefits from a physiological status, in that the coupling within the model incorporates synaptic plasticity by using Hebbian dynamics. The network of neurons, employs the Kuramoto-Sakaguchi model, and adds to it a set of Hebbian synapses. This model, studied here for the first time, reveals emergent chimera states. We study certain characteristics of such chimera states, and also show how the model brings about the possibility of learning and memory.

The non-local coupling used in our model has also been extended to global coupling, or other networks with more complex topology, such as scale free, showing chimera states (Chandrasekar et al., 2014; Zhu et al., 2014). The emergence of chimera states out of arbitrary initial conditions, as we observe in our present model, has similarly been observed in Zhu et al. (2014) in the context of a different model. The network structure can also be such that a pair of coupled populations with time varying interactions is present, and the chimera emerges when the interpopulation links depend on time (Buscarino et al., 2015). In another study, a multilayered network is present, where each layer has an ensemble of non-locally coupled Kuramoto-Sakaguchi identical phase oscillators, and parameter mismatches exist between the layers (Maksimenko et al., 2016).

The question of full synchronization, and how it can be disrupted while keeping the stability of the dynamics is important. Zanette (2005) introduced a frustration function that gives a measure of the competing effects of the attractive and repulsive interactions in a system of oscillators. Even in presence of frustration, where a fraction of couplings are repulsive, full synchronization, keeping all oscillators in phase and pace, can be stable. As the repulsive interaction grows stronger, the synchronized cluster breaks down. Similarly, Rakshit et al. (2019) derived the necessary conditions for the transition from a chimera to coherence by means of a coherent stability function approach.

Frustration can also be introduced through the interactions having a phase frustration. A system of Kuramoto oscillators naturally accommodates a phase frustration, becoming the Kuramoto–Sakaguchi model. It is known that such interactions too, could prevent full synchronization (Kuramoto, 1975; Acebrón et al., 2005). Such Kuramoto oscillators when placed on a complex network, show complicated remote synchronization properties based on the symmetry of the network (Nicosia et al., 2013). Although remote synchronization is preferred based on symmetry of the underlying network, there are other studies that bring the distance function directly into the interaction, and find various possible types of synchronization in presence of mobility of the nodes and frustration of the network (Chowdhury et al., 2019, 2020b). It is possible to tune the synchronization to a single state, for a distribution of natural frequencies, and a distribution of frustration parameters, i.e., phase lags (Kundu et al., 2018).

The relationship between frustration, and topology for the complex network, can determine the final dynamical state, when repulsive or repressive interactions are present, and the frustration function helps in this regard (Levnajic, 2011). Here, the final frustrated state shows multistability. If emphasis is put on the network having a bipartite nature, the presence of repulsive links results in an antiphase synchronization as the final dynamical state (Chowdhury et al., 2020a).

In a similar study to ours, i.e., of non-locally coupled neural oscillators (Sakaguchi, 2006), using a different type of synapse, it was found that the synchronized motion could become unstable, leading to non-uniform states. At a more fundamental level, the chimera state has also been related to the synchronized state of the Kuramoto model *via* a pitchfork bifurcation (Kotwal et al., 2017). A recent study has also concentrated on the interplay of the neuronal dynamics and chimera (Majhi et al., 2019).

To associate learning and memory, we need to first recall that a change in the behavior that comes from experience is the result of learning and memory. The retrieval process completes this association (Thompson, 1986). There are neural mechanisms for learning, and in fact they are proposed as mechanisms of memory too (Eccles, 1964; Woody, 1982). In more advanced theory, there are memory traces in the brain that are required for some forms of learning (Thompson, 2005; Shutoh et al., 2006). This means that a neuronal circuit is involved in the recollection of a particular memory (Mayford et al., 2012). It is then possible to affect learning by changing the strength of the associated memory trace (Li et al., 2020). In our study, it is the notion of frustration that is important for memory, as we discuss in the next section. We eventually associate a local minimum and the neurons involved in it, to the formation of such a trace or circuit. The chimera state comes into play as it relates to the biological state of the array of neurons.

In sum, the present study uses Hebb's synapse to facilitate time dependent neuron coupling in the Kuramoto-Sakaguchi model. We thereby demonstrate the emergence of a chimera state in a biologically meaningful setting. We also show how the incorporation of synaptic plasticity brings about the possibility of learning and memory; the latter, being associated with particular configurations of the time evolving neuron coupling coefficients.

The paper is arranged as follows. Section 2, presents the synaptic model, along with its dynamics, and compares it with some other models. Section 3 is devoted to our results, and how they come about from the unique properties of our model. The main result is the emergence of the chimera state. In Section 3, we discuss the stability of chimera state as a function of frustration, and how the initial conditions become immaterial in the emergence of the chimera state. It is also shown how the presence of a pacemaker brings hysteresis in the dynamics. In Section 4, we conclude by returning to the biological viewpoints based on learning and memory. Intricacies of the computational methods can be found in the Appendix.

## 2. The Model

The model we use is based on Hebb's theory of synaptic plasticity, which can explain learning and memory as being essentially the same thing (Hebb, 1949). According to this view, it is the strength of the synapse connecting two neurons that can represent memory. A fixed graph is a fixed set of neurons and synapses, nodes and links, yet it is the plasticity of this fixed set of synapses that contains the learned message. For a recent review and critique of this viewpoint (see Langille and Brown, 2018).

The synapse has also been identified as the “functional unit of the brain” (Mayford et al., 2012). Synaptic modification forms the basis of memory storage. According to Hebb, the synapse is strengthened if the activity of the presynaptic neuron excites the postsynaptic neuron. This type of synaptic modification occurs in the CA1 region of the hippocampus (Lisman and McIntyre, 2001).

The Hebb rule for the plasticity *c*_*ij*_ of the *ij* synapse is given by


$$\dot{c_{ij}}=\left\{ \begin{array}{l} \;\epsilon (\alpha -c_{ij})e^{-\frac{\text{Δ}\varphi_{ij}}{\tau_{p}}}\text{for Δ}\varphi_{ij}\in [0,\pi ],\text{ (1)}\\ \;-\epsilon c_{ij}e^{\frac{\text{Δ}\varphi_{ij}}{\tau_{d}}}\text{ for Δ}\varphi_{ij}\in (-\pi ,0).\text{ (2)} \end{array}\right.$$

The synapse is modified positively; i.e., strengthened, and can reach the maximum α, as Equation (1) indicates. It also shows that the strength of a synapse, *c*_*ij*_ is activity dependent, and already contains traces of memory. The synapse is weakened; i.e., modified negatively, as Equation (2) shows, and can reach a minimum fixed point of zero.

In the planar rotator model, a rotating unit arrow's location on the plane is determined by its polar coordinate, or phase φ. The model, also known as the XY model, has helped in that the relative phase can determine the relative timing of the presynaptic and postsynaptic spikes, so when a neuron is ahead in spiking, its phase leads. If the postsynaptic spike comes after the presynaptic spike, the synapse is strengthened: Δφ_*ij*_ = φ_*j*_ − φ_*i*_. Here, *j* denotes the presynapse, and *i* the postsynapse, so that when Δφ_*ij*_ > 0, the synapse is strengthened. We have to note that we need activities in both the presynapse, and the postsynapse, if we are to talk about synaptic modification.

The parameters τ refer to the learning time, and the larger they are the faster is the learning. We can see this by first noting that the exponent in Equations (1) and (2) is negative. So the larger τ is, the larger is ċ_*ij*_, in magnitude, and accordingly, the synaptic modification is larger; hence, faster learning. The two times, τ_*p*_ and τ_*d*_, need not be the same, as will be discussed later.

The coupling constant is incorporated within the Kuramoto-Sakaguchi model (Acebrón et al., 2005),


(3) $$\begin{array}{r} \overset{∙}{\varphi_{i}}=\omega_{i}-\frac{1}{2n}\overset{N}{\underset{j=1}{\sum }}c_{ij}\sin\left(\varphi_{i}-\varphi_{j}+\sigma \right), \end{array}$$

where the phases, φ_*i*_(*i* = 1, …, *N*) are for a circular chain of phase oscillators, each having a natural frequency ω_*i*_, and initially identically coupled through the symmetric matrix *c*_*ij*_, with elements either equal to α or zero. As the phases evolve, the coupling matrix elements are developed by the Hebb dynamics. This interactive coupling dynamics is different from that used in traditional chimeras for which the coupling is defined *via* a distance function (Abrams and Strogatz, 2004, 2006; Omel'chenko, 2018; Wang et al., 2019). There is a Kuramoto synchronization study with no relation to chimera that tries to employ a kind of Hebbian coupling, but with no time ordering effect, only giving value to close pre-and-post spikes (Timms and English, 2014).

The time dependent coupling, because of the Hebbian rule, tends to inhibit clustering in the sense of the distance dependence once a complex network is the framework of the Kuramoto model (Nicosia et al., 2013). The coupling, although can be positive and negative, as the Hebbian rule shows, is only effectively so, and again is different from the attractive-repulsive type considered in some other models (Zanette, 2005; Chowdhury et al., 2020b). The reason is that a faster neuron can at times be a presynapse, and at other times act as a postsynapse. This is a complexity that gives the model the potential to accommodate the chimera state.

The Sakaguchi parameter σ, also known as the phase-lag (Panaggio and Abrams, 2015) or frustration (Botha and Kolahchi, 2018) parameter, has a significant effect on the dynamics. This is what we touched upon in the Introduction. In presence of σ, a Lyapunov function for the Kuramoto Hamiltonian, is no longer available, and in the studies of stability, other tools have been developed (Watanabe and Strogatz, 1994; Zanette, 2005; Rakshit et al., 2019). As we have mentioned, Zanette (2005) defines a frustration function, while Rakshit et al. (2019) makes use of a coherent stability function. Unfortunately, neither of these approaches can facilitate an analytic treatment of the present system of Equations (1)–(3), which model a non-locally coupled ensemble of neural oscillators, with each oscillator being time-dependently coupled to its *n* nearest neighbors, i.e., to *n* neurons on either side of it, with *n* > 1 and 2*n* + 1 ≤ *N*.

In a dynamic response, we could seek a synchronized spectrum. It is known that Hebbian dynamics and synchronous response go together to the benefit of the biological system (Cassenaer and Laurent, 2007). Of course, synchronous firing of neurons could also be harmful, as in epileptic seizures. A model with synchronization responses that do not live long could reproduce such seizures (Frolov and Hramov, 2021). Non-locally coupled FitzHugh-Nagumo (FHN) oscillators have applications in neuroscience, and depending on the parameters can give rise to chimera states (Omelchenko et al., 2013). The FHN oscillators can demonstrate chimera state even if the elements are in the excitable mode (Isele et al., 2016). The excitable elements are able to localize the chimera, an aspect which is present in our model too, and which we discuss in the next section. The FHN oscillators on complex networks can model epileptic seizures (Gerster et al., 2020). The chimera states also exist in ensembles of bursting Hindmarsh-Rose neurons, even if the coupling is local (Bera et al., 2016), and in a discrete neuronal model that was recently developed by Khaleghi et al. (2019) to facilitate an analytic treatment without loss of the essential behavior.

We are interested in the dynamics we introduce, as it can model certain aspects of the memory. For this, we need to emphasize that the ground state spectrum of the XY model, in presence of the frustration, σ, develops a complicated texture—the energy landscape acquires a huge near degenerate set (Teitel and Jayaprakash, 1983; Watanabe and Strogatz, 1993). We can imagine an egg crate potential landscape with a fine structure formation, in many scales, on its maxima and minima. The rough terrain in the energy landscape, due to frustration, is what we take advantage of in synaptic modification, to create a vast memory storage. We will show that depending on frustration σ, the steady state pattern of synchrony can shift from a fully synchronized set of neurons, to a chimera pattern. In a chimera pattern, the collection of neurons (oscillators) breaks up into a symmetric set that moves in synchrony, and a set that keeps its asymmetric state. Both sets are simultaneously stable (Abrams and Strogatz, 2004).

## 3. Results and Discussion

The asymmetric pattern that makes a chimera is known to be a stable bifurcation from the symmetric synchronized pattern (Kotwal et al., 2017). We can heuristically think of σ = π/2 as the extreme frustration, and expect the instability to occur close to it.

The Hebb dynamics allows the phases to scatter, as opposed to cluster, meaning that a phase that leads another phase, could lag it as time evolves. This is known to make synchronization likely (Acebrón et al., 2005). The non-local interaction among the phases simply means that we also have interaction between phases that are not nearest neighbors. The learning time now plays a role, and slow learning allows a quasi–adiabatic evolution.

**Figure 1:**
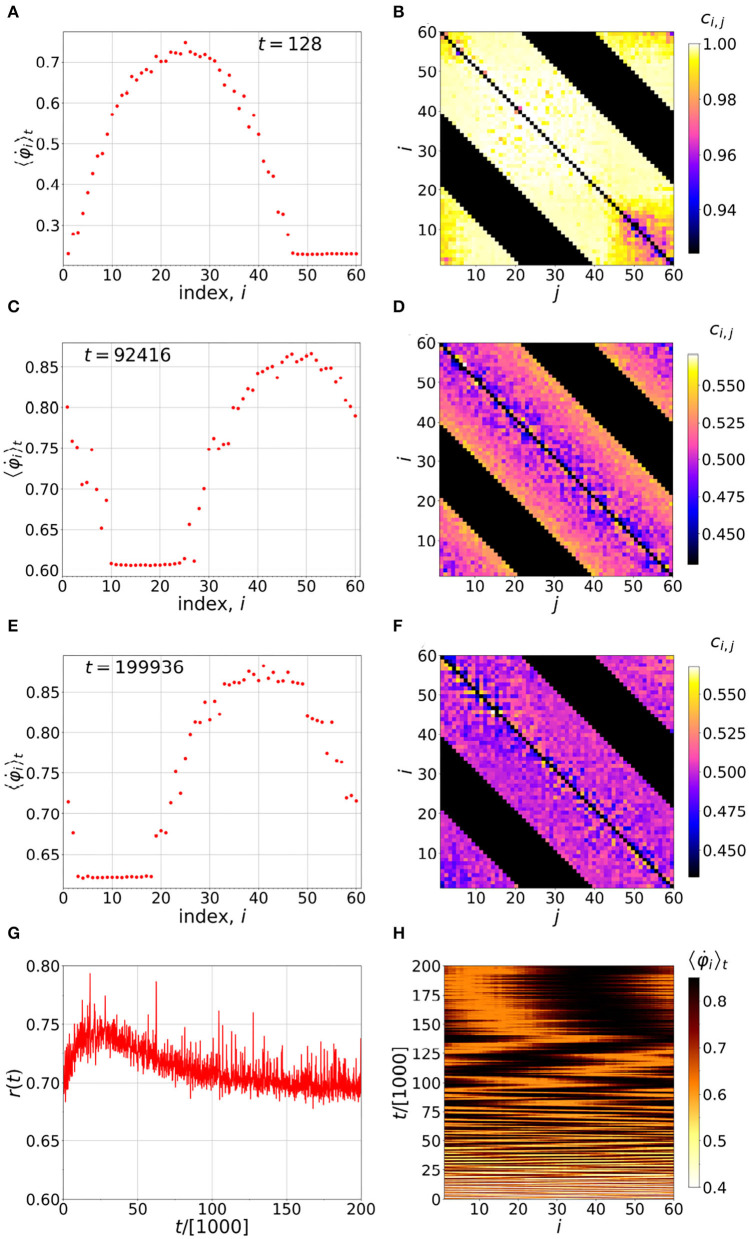
Time evolution of the chimera. **(A,C,E)** Distributions of the running time-averaged firing frequencies of the neurons. **(B,D,F)** Instantaneous coupling strengths at the end of the averaging times indicated in the corresponding figures on the left. **(G)** Time evolution of the order parameter $r\left(t\right)=|\underset{j}{\sum }e^{i\varphi_{j}\left(t\right)}|/N$ and **(H)** distribution of running time-averaged neuronal firing frequencies. Simulation parameters: *N* = 60, *n* = 20, α = 1, τ_*p*_ = τ_*d*_ = 0.001π, σ = 1.478, ϵ = 0.01, and ω_*i*_ = 1 for *i* = 1, …, *N*.

By evolving the system Equations (1-3), as described in the Appendix, we can visualize the time evolution of the chimera, as is shown, for example, in Figure 1. In this instance, *n* = 20, and the black bands seen in Figures 1B,D,F correspond to non-interacting *i* and *j*, i.e., neurons which are more than 20 neighbors apart. The colored bands show the interacting neurons, with the strength of the interaction given according to the color scale. It is interesting how the coherent section creeps in as time evolves, and how it is stabilized next to the incoherent sector. We see a more or less symmetric coupling matrix, with the coherent sector having a slightly less coupling compared to the incoherent sector. In Figure 1H, we can also see non-stationary behavior of the chimera, which is well-known for finite-*N* chimeras, due to their stability properties (Xie et al., 2014; Bera et al., 2017).

Figure 1F also shows that, over long times, the coupling matrix approaches near uniformity and, along with it the wandering motion of the chimera, becomes much smaller. Yet, the character of the chimera is preserved; namely, there is a near synchronized sector, and another moving faster relative to it, not synchronized at all. Hence, at long times we deal with a near traditional chimera where all oscillators are identical, in coupling and otherwise.

In the usual Hebb dynamics, without a chimera state, a uniformly synchronized state is achieved in which the coupling coefficients *c*_*ij*_ are either equal to α or zero, depending on whether the presynapse leads or lags, respectively (Ansariara et al., 2020). In the present system, when the chimera is present, a steady state with small fluctuations in the coupling coefficients is reached. However, for this steady state the average quantity, (Σ_*i, j*_*c*_*ij*_)/(2*nN*), is constant, as we will see, shortly. In fact, these steady state fluctuations in the *c*_*ij*_ may play a role that is analogous to thermal fluctuations on molecular dynamics (Rapaport, 2004) or, more generally, any type of noise, as we will discuss at the end of Section 3.

In our earlier study (Ansariara et al., 2020), which did not involve chimera states, we showed that for a symmetric synapse where, τ_*p*_ = τ_*d*_, the time averaged coupling is α/2. This came about as we investigated a bistability, which was essential for the Lisman switch, and remained time–symmetric for the time–symmetric synapse.

**Figure 2:**
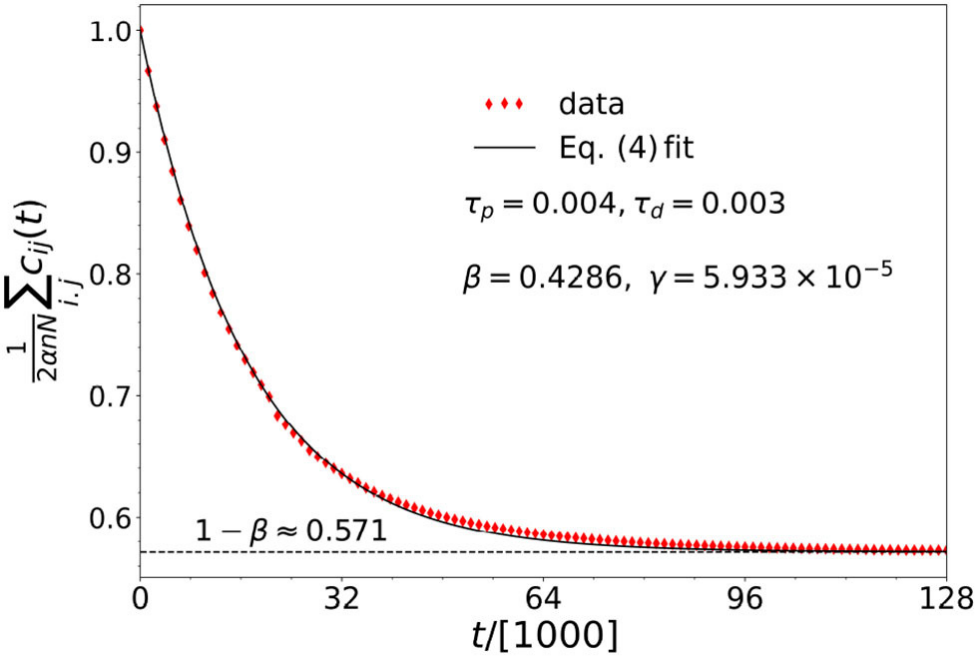
The best fit (*R*^2^ = 0.998) provided by Equation (4) (solid black line), to the numerical data (red diamond-shaped markers), for the average of the coupling matrix elements as a function of time. See the main text surrounding Equation (4) for details. Here, the parameters are: *N* = 40, *n* = 14, α = 1, σ = 1.478, ϵ = 0.01, and ω_*i*_ = 1 for *i* = 1, …, *N*.

In the presence of a chimera state, the average coupling matrix element decays exponentially in time, according to the equation


(4) $$\begin{array}{r} \frac{1}{2\alpha nN}\underset{i.j}{\sum }c_{ij}\left(t\right)=1-\beta +\beta \exp\left(-\gamma t\right), \end{array}$$

where β = τ_*d*_/(τ_*p*_ + τ_*d*_). The coupling matrix elements thus all tend toward the average 1−β. This is an empirical finding as shown in Figure 2. Here, the average coupling matrix element has been normalized to α. This result matches our earlier result of α/2 when the two learning times are equal.

As we have seen in Figure 1, with a slow learning time, a long time is required to reach the steady state. We emphasize here that, in our dynamics, even after reaching the steady state, the elements of the coupling matrix are not all constant and equal, as would be the case for a traditional chimera. Nevertheless, as Figure 1H shows, the chimera is non-stationary, meaning its position fluctuates with time. Such non-stationary behavior is well-known from finite *N* studies of traditional chimeras, and it can be described well by Brownian motion (Wolfrum et al., 2011; Omel'chenko, 2018).

The spatiotemporal configuration of the chimera changes, as Figure 1 indicates. This means that oscillators that run faster on average pass each other, and take each other's place. If such an overall motion of the chimera is to be localized, we need a situation that does not allow this kind of overtaking. According to the Hebb rule, Equations (1)–(2), if an oscillator's motion; i.e., a neuron's firing rate, is fast enough relative to the neurons it is directly in contact with, the other neurons must follow it, and hence its coupling will not be affected. This makes a barrier in that the neighboring oscillator cannot overtake the leading neuron, as if a “fixed boundary condition” were imposed at the site of the leading oscillator. This particular neuron is then called a *pacemaker* (Ansariara et al., 2020).

**Figure 3:**
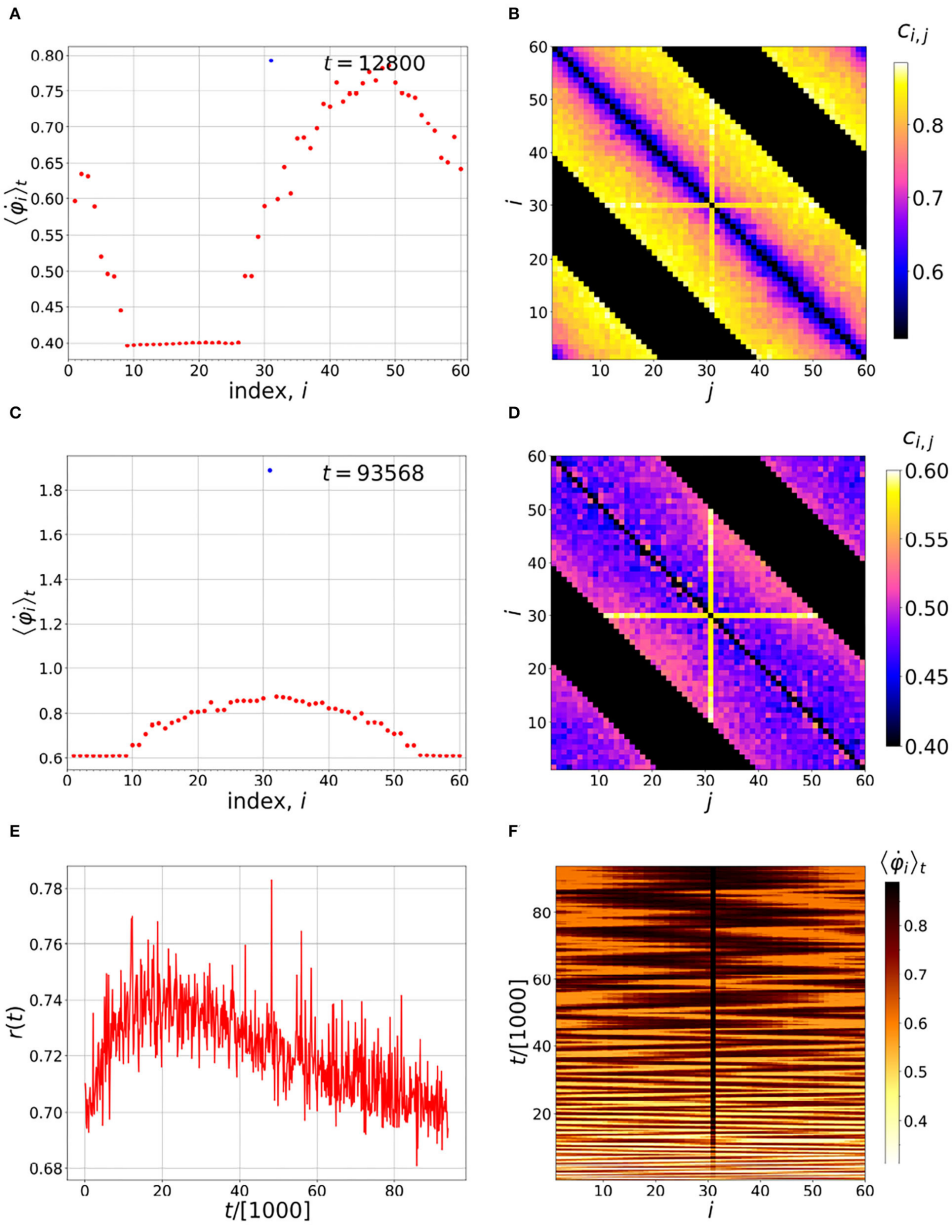
Time evolution of the chimera with a pacemaker at position *i* = 31. **(A,C)** Distributions of the running time-averaged firing frequencies. **(B,D)** Instantaneous coupling strengths at the end of the averaging times indicated in the corresponding figures on the left. **(E)** Time evolution of the order parameter *r*(*t*) and **(F)** distribution of running time-averaged neuronal firing frequencies. As can be seen in **(F)**, the pacemaker diminishes the wandering of the chimera over time, eventually maintaining the position of the incoherent part of the chimera near *i* = 31. Simulation parameters: *N* = 60, *n* = 20, α = 1, ω_*i*_ = 0 (except *i* = 31), σ = 1.478, τ_*p*_ = τ_*d*_ = 0.003 and ϵ = 0.01.

In Figure 3 there is a pacemaker added *via* *i* = 31, shown by the cross in Figures 3B,D. The natural frequency of this neuron, ω_31_, is slowly increased over time. In the time evolution, there is still a chance for other neurons non-locally coupled to it, to get ahead, affecting it by changing its coupling. But overall, and over long times, the chimera can be controlled by neuron 31, as Figures 3E,F indicate. We note that, in the past, a variety of different control mechanisms have been used on chimera states (Sieber et al., 2014; Gambuzza and Frasca, 2016). There is a similar influence to our pacemaker, coming from excitable FHN elements, and resulting in effective barriers in controlling the position of a chimera state (Isele et al., 2016).

**Figure 4:**
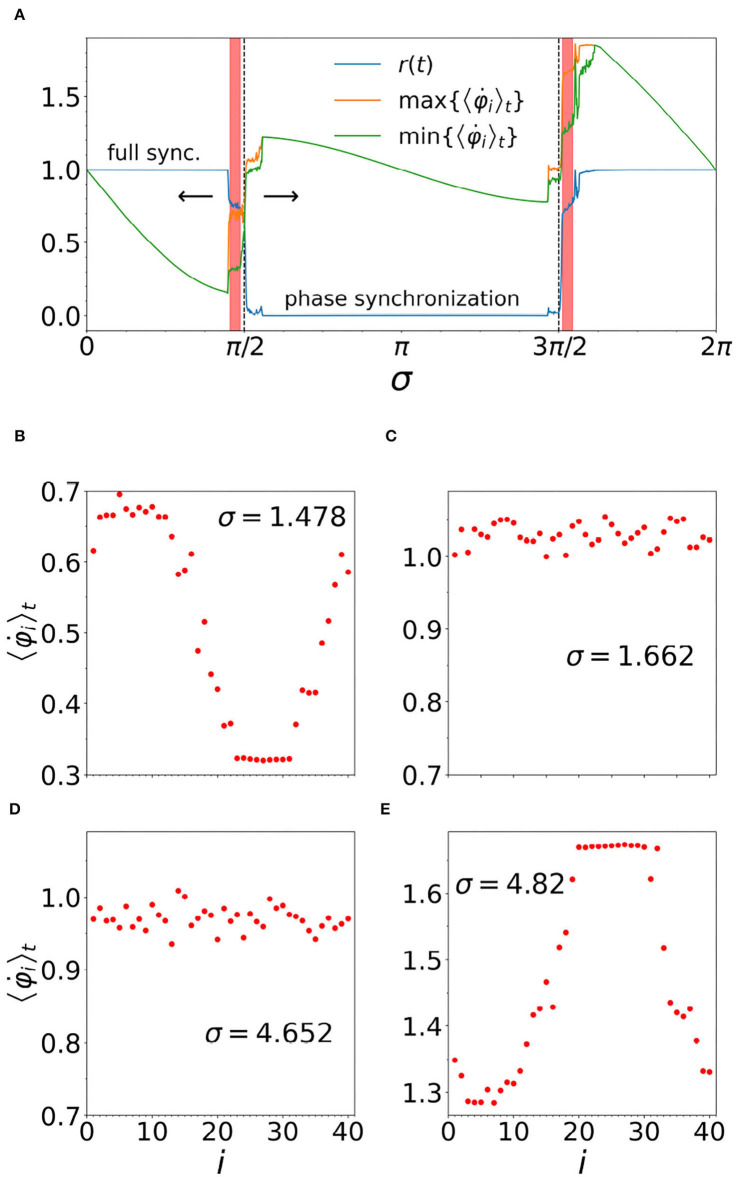
**(A)** Intervals of frustration (σ) leading to stable chimeras, as indicated by the pink shading: σ ∈ (1.43, 1.55) for left interval and σ ∈ (4.75, 4.86) for the right. *r*(*t*) is the order parameter (blue line). Also plotted on the y-axis (labels omitted) are the maximal and minimal average frequencies, as indicated by the legend. Note that, when *r*(*t*) = 1, we have full synchronization, while $\max\left\{\langle {\overset{{}^{\circ}}{\varphi }}_{i}\rangle_{t}\right\}=\min\left\{\langle {\overset{{}^{\circ}}{\varphi }}_{i}\rangle_{t}\right\}$ indicates phase synchronization. Other parameters are: *N* = 40, *n* = 14, α = 1, ϵ = 0.01, τ_*p*_ = 0.003, τ_*d*_ = 0.004, and ω_*i*_ = 1 for *i* = 1, …, *N*. In **(B,E)**, the two different chimera patterns discussed in the main text are shown at representative values of σ. In **(C,D)**, we see examples of the partially synchronized states that separate the two chimera intervals from the large phase synchronous interval, for which all the oscillators rotate at the same frequency, while maintaining a fixed phase difference relative to one another.

Returning to the character of frustration σ in the rough texture of the ground state space, in Figure 4, we investigate the stability of the chimera pattern of synchrony, as a function of the frustration. Here, we start from a chimera at σ = 1.478 and slowly increase (decrease) the value of sigma, as indicated by the arrows. The chimera is stable in a narrow vicinity to the left of the dashed vertical line shown at σ = π/2, and toward the right of the dashed vertical line at 3π/2. This result is in agreement with the analytical study on the subject (Kotwal et al., 2017).

Comparison of Figures 4B,E shows that, for chimeras just below σ = π/2, the coherent region runs slower than the incoherent region, while for the chimeras just above 3π/2 it is the opposite, i.e., we have an “upside down” chimera, as this comparison clearly shows. We also note that as σ increases through the large phase synchronous region surrounding σ = π, the upside down chimeras form spontaneously from the random configuration of phases, once σ reaches the range of stability. Similarly, we have found that spontaneous formation of the chimera in the left hand range of stability also occurs if σ is slowly decreased, starting from a completely incoherent phase synchronous state at, say, σ = π. Thus the chimeras discussed in our present work do not require any special initial conditions (cf. Section 3 of Abrams and Strogatz, 2006).

**Figure 5:**
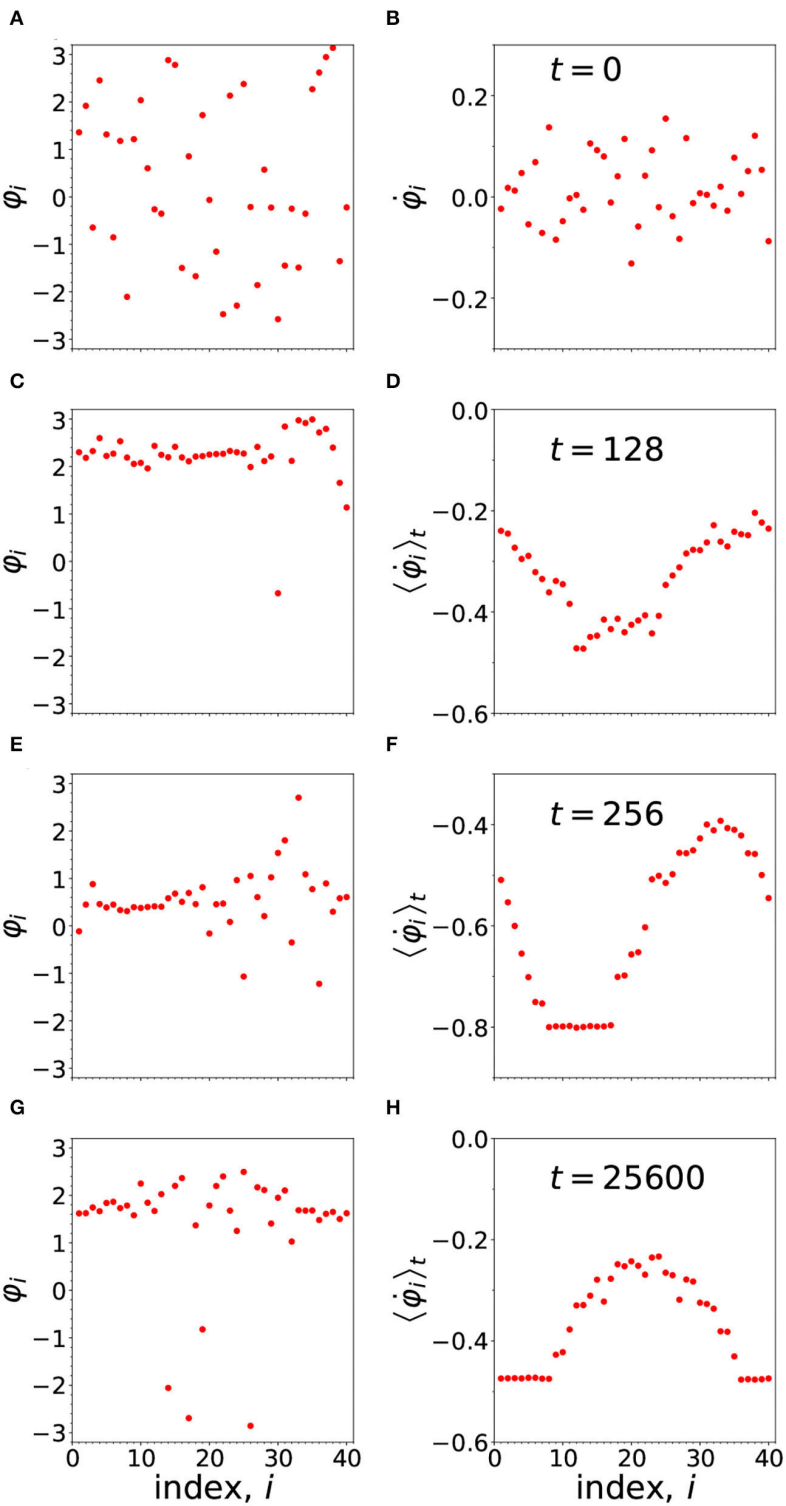
The development of the chimera pattern from the arbitrary (random) initial configuration of phases shown in **(A)**. The figures to the left of the panel **(A,C,E,G)** show the instantaneous phases at times *t* = 0, 128, 256, and 25,600, respectively. On the right hand side (in **B,D,F,H**) are shown the frequencies. In the case of **(B)** we plot the initial instantaneous frequency, while in **(D,F,H)** we plot the running average frequencies, computed as described in the last paragraph of the Appendix. The parameters are: *N* = 40, *n* = 14, α = 1, ω_*i*_ = 0, σ = 1.478, τ_*p*_ = τ_*d*_ = 0.003, and ϵ = 0.01.

In Figure 5, we have an example of a chimera, produced from a random set of phases, which means, a random set of synaptic strengths. As time evolves, following the dynamics we have introduced, a chimera pattern of synchrony sets in, defining a particular memory.

**Figure 6:**
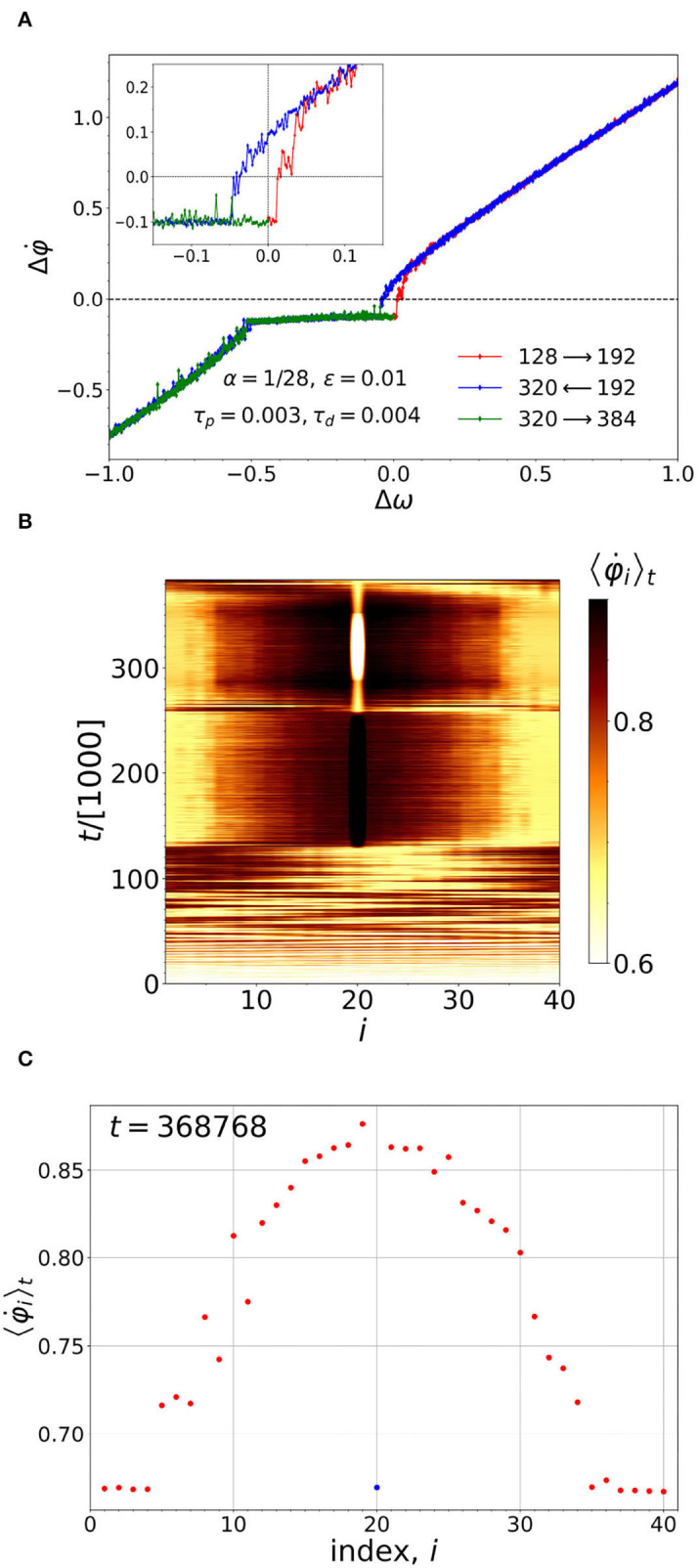
**(A)** The pacemaker leading the chimera around a hysteresis loop (see main text for details). The inset shows a close-up view of the hysteresis loop. **(B)** Before the pacemaker is turned on, the chimera moves about randomly throughout the oscillators. After the pacemaker is turned on at *t* = 128, 000, the position of the chimera becomes correlated with that of the pacemaker. **(C)** The configuration of the chimera near the end of the cycle, shown in **(A)**. The pacemaker and the coherent part of the chimera are moving in synchrony.

The idea of the pacemaker naturally leads to another question: if the chimera is led by a pacemaker over the energy terrain, will its motion become history dependent? Figure 6 shows that the result of such an experiment is in the affirmative. Initially, in Figure 6A, all the natural frequencies are set to ω_*i*_ = 1. At *t* = 128000, after the system has reached its steady state, the pacemaker neuron, *i* = 20, is turned on by slowly increasing its natural frequency above ω = 1 (as described in the last paragraph of Section 3). The difference, Δω = ω_20_−1, is plotted along the x-axis. On the y-axis, we plot the difference between the running average $\langle {\overset{∙}{\varphi }}_{20}\rangle_{t}$ for the pacemaker, and the average frequency, averaged over the rest of the ensemble (excluding the pacemaker). The complete cycle then consists of (i) increasing Δω from zero to 1 (red curve), (ii) decreasing Δω from 1 to -1 (blue curve), and (iii) increasing Δω back up to 0 (green curve). In so doing the hysteresis loop is formed.

The hysteresis appearing in our system is likely due to bistability or multistability in the dynamics, similar to that reported in Belykh et al. (2016) for a system of Kuromoto oscillators with inertia. However, to investigate the details of this dynamics in our system is not a simple matter. Due to the presence of the frustration parameter in our system, the hysteresis can obviously not be understood in the usual way, i.e., in terms of a potential energy barrier that traps the system as the stimulus is changed. Thus, to provide a rigorous mathematical classification of the underlying bifucations one would have to follow an alternative, albeit mean field approach. Recently, for example, Dixit et al. (2021) has developed such a mean field approach that facilitates the use of sophisticated numerical continuation software (XPPAUT), to obtain the full bifurcation structure. A similar analysis of the present system could be a fruitful topic for future research. The hysteresis we observe may also be related to a recently reported phenomenon called self-adaptation of chimera states (Yao et al., 2019), and this possible connection could also be explored in the subsequent work. At this juncture, we simply present the numerical evidence that the pacemaker in effect is reshaping the energy terrain, and guiding the array of neurons in some way.

Discussion of memory and learning is naturally linked with the complex set of potential barriers. The cognitive state may be characterized as being collectively purposefully non-stationary. This may seem as a trivial characterization, in light of all the molecular changes underlying long-term synaptic plasticity, and its role in memory storage. It was discovered that the many forms of synaptic plasticity are in fact required in the persistence of memory (Mayford et al., 2012). So we think of a time dependent collective state, with a time dependence that is guided in a complex manner, defining procedural and declarative learning, as a result of changes in synaptic strengths. There are specific Hebbian learning rules that establish synaptic sequences needed for storage and retrieval, similar to the dynamics observed in hippocampus and parietal cortex (Gillett et al., 2020).

In our model of arrays of neurons connected with Hebb synapses, the XY model helps in this dynamics, moving the state in the vast space of local minima, thanks to frustration. We can associate a fully synchronized state to a particular memory, yet this does not play well with the neurophysiological mechanisms. A chimera state in our set of neurons can simulate a memory trace. In other words, we associate the chimera state of neurons that have come about by the appropriate collectively modified synapses, to a memory circuit. This state is not stationary in time, just as a cognitive state is not. The fully synchronized state is a dislocated cognitive state, as in a seizure. Hence, the collective simultaneity of the coherence and incoherence as present in a chimera, keeps the cognitive state in order, as the basis of various types of memory and learning.

In addition, there is an unexpected result: in Figure 6A we see a plateau-like region which is due to the partial synchronization of the frequencies of the oscillators of the coherent cluster by the leading oscillator (peacemaker). Figure 6C shows the time averaged frequencies of all the oscillators corresponding to Δω = −0.2437 (increasing) within the plateau-like region. It shows that, on the plateau, the average frequency of the pacemaker is the same as that of the coherent region.

By way of discussion, we mention that this behavior is reminiscent of the resonance that occurs in a system of LC shunted Josephson junctions, in which the Josephson frequency can be controlled *via* the dc-bias current to resonate with the natural frequency of the shunting circuit, giving rise to a so-called resonance circuit branch (rc-branch) (Shukrinov et al., 2015). Such resonance tends to synchronize the Josephson junctions. Here, the pacemaker oscillator appears to act like the resonance circuit in the sense that it causes the partial synchronization of the oscillators in the coherent cluster by the same type of “frequency pulling” mechanism that is well-know in many non-linear systems (Hilborn, 2000). Note that, unlike Shapiro steps, which occur in the driven Josephson junction due to frequency locking with the external excitation, the rc-branch is not completely flat, like the plateau seen here.

Furthermore, when the pacemaker frequency becomes too different from that of the coherent part, we also observe a transition off the plateau, which is akin to reaching the ends of the rc-branch. In fact, this behavior of the current system may also be related to that of coherence-resonance chimeras, as originally reported in Semenova et al. (2016). In the latter work the locations of coherent and incoherent domains is also observed to interchange, much like we saw in Figures 4B,E. Moreover, in our case the fluctuations in the *c*_*ij*_, brought about by the Hebb rule, may play an analogous role to the noise in systems exhibiting coherence-resonance chimeras. In future work we intend to investigate these questions in greater detail.

## 4. Conclusion

We can think of the chimera as a harmonious pattern, whose structure within, serves a function that the uniform synchronized state could not. We can study the brain with the special eye on how a chimera state can help in organization of the cognitive state (Bansal et al., 2019).

In the model we studied, with the synapses being of the Hebb type, we discovered that the coupling or the strength of the synapse goes through an evolution. As the synapses are modified, the collection learns. But eventually, upon reaching the steady state, all synapses are statistically in the same modified state, and memory is lost. Although we have a memory that forgets, the approach to the modified state of the synapses could be designed to take an arbitrarily long time. In this way, the chimera could give a more advanced learning/memory.

## Data Availability Statement

The raw data supporting the conclusions of this article will be made available by the authors, without undue reservation.

## Author Contributions

AEB and MRK conceptualized the idea for this work. MA performed trial simulations using a C++ code and contributed to discussions. SE contributed as a domain expert to biological aspects of the work. AEB performed all the final numerical simulations and produced the figures. All the authors contributed equally to the writing of the original manuscript, its review, and final editing. All authors approved the submitted version.

## Conflict of Interest

The authors declare that the research was conducted in the absence of any commercial or financial relationships that could be construed as a potential conflict of interest.

## Publisher's Note

All claims expressed in this article are solely those of the authors and do not necessarily represent those of their affiliated organizations, or those of the publisher, the editors and the reviewers. Any product that may be evaluated in this article, or claim that may be made by its manufacturer, is not guaranteed or endorsed by the publisher.

## Figures and Tables

**Figure A1:**
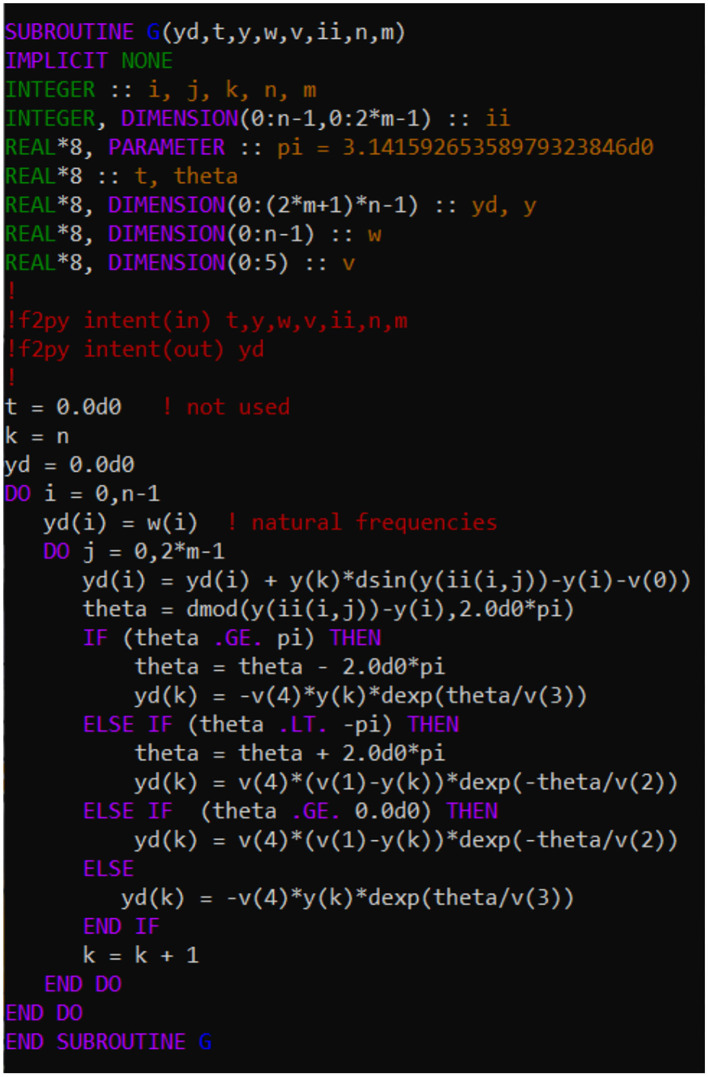
Fortran 90 code for returning the vector of first derivatives for the system of Equations (1)–(3). The parameters are stored in the vector *v* in the order σ, α, τ_*p*_, τ_*d*_, and ϵ. The variable *theta* corresponds to Δφ_*ij*_ in Equations (1)–(2).

**Figure A2:**
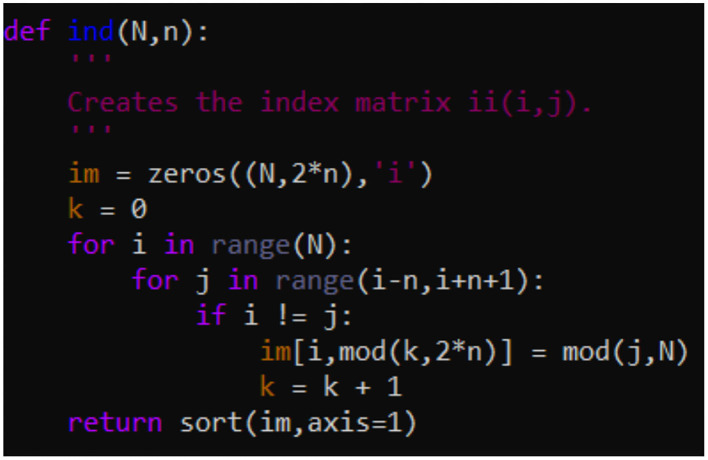
Python function for generating the index matrix *ii* that is passed *via* the 6th argument into the Fortran 90 subroutine shown in Figure 1.

## Appendix: Computational Methods

Equations (1)–(3) constitute a set of (2*n*+1)*N* coupled differential equations. Even for *N* = 40 and *n* = 14, (2*n*+1)*N* amounts to more than a thousand equations, making it time consuming to solve. It thus becomes essential to set up and solve Equations (1)–(3) in a way that is computationally efficient. In the present work we have used a convenient and efficient combination of Fortran 90 and Python 3 codes. To benefit from the greater computational efficiency of Fortran 90, compared to Python, we coded a Fortran 90 subroutine for returning the first derivatives. In this subroutine we use a Python generated index matrix *ii* to stack only the 2*nN* non-zero components of the adjacency matrix into the returned vector of first derivatives. For convenience we list the Fortran 90 code for returning the first derivatives in Figure A1. Figure A2 gives the python function for generating the index matrix *ii* that is passed into the Fortran subroutine.

To solve the system we first wrap the Fortran subroutine into a Python callable function using f2py and then employ the Dormand–Prince routine dopri5 (Hairer et al., 1993) from the Python module scipy.integrate (Virtanen et al., 2020). In dopri5 we set both the relative and absolute integration error tolerances to 10^−12^. At these settings the code uses approximately 0.427[(2*n*+1)*N*]^1.704^ CPU seconds to evolve the system by 100 000 time units and is available online: https://doi.org/10.24433/CO.9507305.v1.

In Figure 2, we obtained the best fit parameters for Equation (4) by using the Python routine curve_fit, imported from scipy.optimize (Virtanen et al., 2020), with the default optimization method, leastsq.

For the running averages, such as those plotted in Figures 1A,C,E,H, we have averaged over 128 time units, sampling every 0.5 time units. When we apply a pacemaker, as shown in Figure 3, for example, we change the pacemaker's natural frequency, ω_*i*_ (for some fixed *i*), by 5 × 10^−5^ every 0.5 time units.
